# DNA Polymerase Beta Catalytic and Fidelity Mutations Drive Platinum-Specific Drug Sensitivity

**DOI:** 10.3390/cancers18162531

**Published:** 2026-08-07

**Authors:** Jacob Lindquist, Josh Heyza, Istri Ndoja, Nasrin Movahhedin, Chris Yunker, Marina Cardo-Vila, Seongho Kim, Joann Sweasy, Steve M. Patrick

**Affiliations:** 1 Department of Oncology, Wayne State University and Karmanos Cancer Institute, Detroit, MI 48201, USAmovahhedinn@karmanos.org (N.M.);; 2 Department of Pharmacology, Institute of Environmental Health Sciences, Wayne State University and Karmanos Cancer Institute, Detroit, MI 48201, USA; 3 Department of Otolaryngology, University of Arizona, Tuscon, AZ 85724, USA; 4 Eppley Institute for Research in Cancer and Allied Diseases, University of Nebraska Medical Center, Omaha, NE 68198, USA; jsweasy@unmc.edu

**Keywords:** DNA polymerase β (Polβ), base excision repair (BER), cisplatin, DNA repair, interstrand crosslinks, nucleotide excision repair (NER)

## Abstract

**Background/Objectives**: With the advent of genome sequencing and its widespread use in the clinic, there is a great need to identify mutational biomarkers that predict therapeutic responses. DNA polymerase beta (Polβ) and the base excision repair (BER) pathway have been previously implicated as modulators of response to platinum-based chemotherapies and are mutated in as many as 30% of cancers. **Methods**: Here, we show in a triple-negative breast cancer (TNBC) model that two classes of mutations in Polβ, reduced catalytic activity (E295K and D256A mutations) and reduced fidelity (I260M), are sufficient to drive cisplatin and carboplatin-specific sensitivity. Cellular response to oxaliplatin in these Polβ mutant models is minimal relative to cisplatin and carboplatin. **Results**: We show that sensitivity is associated with reduced repair of both platinum-induced DNA intrastrand adducts and interstrand crosslinks (ICLs). Downregulation of the upstream BER factor uracil DNA glycosylase (UNG) reverses drug sensitivity, which is consistent with these Polβ mutations negatively impacting ICL DNA repair to drive drug sensitivity. In addition, the intrastrand adduct repair readout indicates that these lesions also play a role in the sensitivity observed in Polβ mutant models. In vivo studies demonstrate a significant effect on tumor growth delay with cisplatin treatment in tumor xenografts harboring Polβ mutations. **Conclusions**: These results support the potential for using Polβ mutations as predictive biomarkers for cisplatin and carboplatin therapies in the clinical setting.

## Simple Summary

DNA polymerase beta (Polβ) is a key protein for base excision repair (BER) and synthesizes nucleotides during the repair of DNA abasic sites. Previous work has demonstrated that loss of Polβ activity can drive resistance to cis/carboplatin chemotherapy. In many cancers, Polβ is mutated in the protein domains responsible for its catalytic polymerase activity. These cancer mutations in Polβ can impact both the catalytic efficiency as well as the ability of Polβ to incorporate the correct nucleotide (fidelity activity). There is some work that has shown that specific Polβ mutations can result in sensitivity to platinum-based chemotherapies, but the mechanisms driving this sensitivity are unclear. In this study, we sought to develop novel triple-negative breast cancer (TNBC) models of two clinically relevant Polβ mutations, E295K and I260M, as well as a catalytic dead mutant, D256A, to assess the impact on platinum-based chemotherapy. E295K, D256A and I260M Polβ mutations resulted in significant sensitization to cis/carboplatin. The sensitization of Polβ E295K and D256A mutants to these drugs correlated with deficiencies in the repair of both intrastrand adducts and interstrand crosslinks (ICLs). In addition, there was a significant tumor growth delay in both E295K and D256A mutants relative to wildtype controls when treated with cisplatin.

## 1. Introduction

Platinum-based chemotherapies are among the most commonly used treatments in cancer, treating patients with cancer types such as ovarian, lung, head and neck, and breast tumors [1,2,3]. These drugs target and bind to DNA, resulting in inhibition of DNA replication and transcription, which can lead to apoptosis [4]. However, recurrence and resistance to these drugs are commonly seen in the clinical setting, highlighting the need to better predict patient response. Multiple mechanisms of cisplatin resistance have been previously described, including decreased drug accumulation, increased drug inactivation, as well as alterations in DNA repair [5]. Increased DNA repair has been shown in cell culture models as well as in patient response to cisplatin, and it is seen in nearly all cases of high cisplatin resistance [4].

Platinums directly bind to DNA, resulting in the formation of mono-adducts, intrastrand adducts, as well as interstrand crosslinks (ICLs) [6,7]. These adducts distort the DNA, leading to fork stalling during DNA replication and transcription and eventual apoptosis if the cell is unable to process or repair these platinum-induced adducts. The main DNA repair pathway that resolves intrastrand adducts is the nucleotide excision repair (NER) pathway, which can also work in conjunction with the homologous recombination (HR) and Fanconi Anemia (FA) pathways to remove ICLs [5,8]. Unsurprisingly, increased activity of these DNA repair pathways has been associated with chemoresistance to cisplatin [9]. Despite understanding the major pathways that repair platinum–DNA adducts, the impact of other pathways as well as various clinically relevant mutations in additional DNA repair factors on cisplatin adduct repair is incompletely understood.

Although all platinum reagents are able to bind to DNA, the intrastrand adducts and ICLs formed by oxaliplatin versus cis/carboplatin differ in the complex structures and distortions induced in the DNA double helix. In particular, the ICLs formed by cis/carboplatin at GpC sites and the covalent link between guanine bases on complementary DNA strands result in the flanking cytosines being extrahelical and flipped away from the duplex structure, which is distinct from the ICLs formed by oxaliplatin [10,11,12,13,14]. Loss of BER as well as mismatch repair (MMR) drives a cis/carboplatin resistance, and these two pathways work in an epistatic manner to mediate cis/carboplatin response [15]. The extrahelical cytosines adjacent to the ICL produced by cis/carboplatin can be de-aminated by specific APOBEC3 family members, after which uracil DNA glycosylase (UNG) is able to generate an abasic site [15]. AP endonuclease (APE1) is then able to cleave the 5′ end of the abasic site, allowing for the recruitment of Polβ to incorporate nucleotides. In the case of nucleotide misincorporation by Polβ, downstream MMR MutSα (MSH2-MSH6) can bind and continue to block ICL DNA repair. Overall, this process results in non-productive processing of the platinum ICL, resulting in persistence of ICL lesions and sensitivity to cis/carboplatin [15,16,17].

Polβ is the main DNA polymerase involved in short-patch BER and is mutated in up to 30% of tumors including lung, colon, gastric, and breast cancers [18,19]. Several of these mutations have been previously characterized and shown to trigger cellular transformation as well as induction of both resistance and sensitivity to platinums based on varying phenotypes [20,21,22]. Previous knockdown of Polβ was shown to induce a cisplatin-resistant phenotype both in in vitro and in vivo models, while expression of a catalytic-deficient mutant form of Polβ in these same models induced a platinum hypersensitivity [15,16,17,22]. Previous work suggested that APOBEC3 family member expression is an important variable that modifies how loss of specific Polβ mutations impact platinum-based chemotherapy responses, especially with regard to the impact on ICL non-productive repair [15,16,17].

Breast cancer remains the most commonly diagnosed and second most lethal cancer among women in the U.S. [23]. In part, this is due to the triple-negative subtype (TNBC), which unlike other subtypes, still lacks targeted therapies. Recent research has highlighted a promising role for platinum use in the treatment of TNBC [24]. However, there remains a large heterogeneous response [25], highlighting the need for predictive biomarkers to help determine the best treatment option. This need for predictive biomarkers alongside the heterogeneous response to platinum among various Polβ mutations highlights the need for further investigation into specific Polβ mutations that drive platinum sensitivity. In this study, we investigate the ability of catalytic-deficient Polβ to induce hypersensitivity to cis/carboplatin. We show through multiple mutations (E295K, D256A and I260M), that catalytic deficiency as well as reduced Polβ fidelity result in platinum sensitivity in a cis/carboplatin-specific fashion. Importantly, this work highlights the contribution of both intrastrand adducts and ICLs in driving the sensitive phenotype in these Polβ mutant models. These results highlight the potential role of specific Polβ mutations in modulating platinum–DNA adduct repair, highlighting their potential utility as predictive biomarkers for predicting platinum response in the clinical setting.

## 2. Materials and Methods

### 2.1. Cell Culture

All cells were maintained at 37 °C in a 5% CO_2_ incubator. MDA-MB-231 (HTB-26) cells sourced from American Type Culture Collection (ATCC; Manassas, VA, USA), were grown in RPMI (Dharmacon, Lafayette, CO, USA) containing 10% fetal bovine serum (FBS) and 1% penicillin/streptomycin (Dharmacon). SUM1315 (HUMANSUM-0003018) cells sourced from Asterand (now BiolVT, Hicksville, NY, USA) were grown in F12/DMEM containing 10% FBS and 1% penicillin/streptomycin. All cell lines were authenticated by the Karmanos Cancer Institute (KCI) Biobanking and Correlative Sciences Core prior to experiment initiation. Cells were tested for Mycoplasm contamination and used within 10 passages for all experiments. The Cellosaurus accession number for MDA-MB-231 cells is CVCL_0062 and the accession number for SUM1315 is CVCL_5589.

### 2.2. shRNA Transfection and Stable Knockdown Generation

MDA-MB-231 cells knocked down for WT Polβ and re-expressing mutant D256A were generated previously (provided by the Robert Sobol lab, Brown University, Providence, RI, USA) [16,17]. Other knockdowns were generated via Mission shRNA plasmid DNA. Lentiviral particles were generated using HEK293T cells using the following plasmids: PMD2G, PMDLG/RRE and PRSV/RRE. Lipofectamine 3000 (Invitrogen, Carlsbad, CA, USA) was used to transfect plasmid DNA. At 24 h post-transfection, the media was changed to media of target cells. Viral particles were harvested and centrifuged 72 h post-transfection before filtration through 0.2 μm filters. Viral stocks were aliquoted and used fresh or stored at −80 °C. Polybrene was added to viral media immediately prior to transfection to maximize efficiency. Transfected cells were selected and stable knockdowns were maintained using puromycin (Sigma-Aldrich, St. Louis, MO, USA) or G418 (Sigma-Aldritch, St. Louis, MO, USA).

### 2.3. Quantitative Real-Time PCR

Cells were harvested and pelleted before extracting RNA via TRIzol (Invitrogen, Carlsbad, CA, USA) as previously described [15]. RNA concentration was quantitated using a nanodrop spectrophotometer, and cDNA was derived from 2 μg of RNA using the High-Capacity cDNA Reverse Transcription Kit (Applied Biosystems, Foster City, CA, USA). Real-time PCR was performed in technical triplicate using PowerUP SYBR Green Master Mix (Applied Biosystems, Foster City, CA, USA), with GAPDH as an endogenous control. Relative gene expression was determined with the 2ΔCT method and performed in biological triplicate as previously described [16,17]. Primer sequences were as follows (Table 1):

### 2.4. Colony Survival Assay

A total of 300–800 cells were seeded into 60 mm dishes one day prior to treatment. Cisplatin (Sigma-Aldritch, St. Louis, MO, USA), carboplatin (Sigma-Aldritch, St. Louis, MO, USA), and oxaliplatin (Sigma-Aldritch, St. Louis, MO, USA) stock concentrations were prepared at 1 mM in phosphate-buffered saline (PBS). Once drug was completely dissolved via vortexing, solutions were filtered through a sterile 0.2 μm filter. Cisplatin and carboplatin stock solutions were prepared fresh for each experiment. Oxaliplatin and methyl methanesulfonate (MMS, Sigma-Aldrich, St. Louis, MO, USA) stock solutions were stored at −80 °C and −20 °C, respectively, following preparation and used within a 6-month period. For cisplatin, carboplatin, and oxaliplatin, cells were incubated with varying concentrations for 2 h in serum-free medium. For MMS, cells were treated in serum-containing medium for 4 h. For the methoxyamine (MX; purchased from Sigma-Aldrich) pretreatment, we followed previously established conditions [15]. Following treatment, drug media was removed and replaced with complete medium and colonies were allowed to grow for 7 days. Plates were stained and fixed with crystal violet solution (20% ethanol, 1% *w*/*v* crystal violet). Plates were counted for colonies with ≥50 cells and percent survival was determined using the ratio of the number of colonies in treated plates compared to the average number of colonies in 0 concentration plates multiplied by 100. Experiments were performed in biological and technical triplicate. IC50 values for each replicate were calculated with effect values at each concentration using CompuSyn (version 1.0, ComboSyn, www.combosyn.com).

### 2.5. MTS Viability Assay

~2000 cells were seeded per well into a 96-well plate one day prior to treatment. Cells were treated with cisplatin for 2 h in serum-free medium at working concentrations diluted from stock solutions prepared as for colony assays. After treatment, drug medium was removed and cell viability was measured 48 h post-treatment. For MTS assays, 10 μL of respective reagent was added to each well and allowed to incubate at 37 °C for 2–4 h. Plates were read at 450 nm (MTS) and percent survival was determined as a ratio compared to the average reading at 0 concentrations.

### 2.6. Modified Alkaline Comet Assay for Cisplatin Interstrand Crosslinks

Modified alkaline comet assay was used to analyze the presence and repair of ICLs as previously described [15,16,17]. Cells were treated with cisplatin (10 μM) for 2 h and collected at 0, 24, 48, and 72 h post-treatment. Immediately prior to collection, cells were incubated with fresh hydrogen peroxide (100 μM) for 15 min to induce DNA strand breaks or control buffer. The use of hydrogen peroxide to induce strand breaks serves as a way to fragment DNA and is a comparative control in measuring ICLs present. The presence of ICLs covalently links the DNA strands, thus retarding migration through the gel [15,16,17]. Once cells were collected, suspensions containing ~10,000 cells were embedded in 0.5% low melting point agarose and pipetted onto a slide pre-coated with 1% agarose. Slides were incubated at 4 °C in lysis buffer (2.5 M NaCl, 10 mM Tris, 100 mM EDTA, pH 10, 1% Triton X-100) for 1 h. After lysis, slides were incubated at 4 °C in alkaline electrophoresis buffer (300 mM NaOH, 1 mM EDTA, pH > 11) for 20 min. Electrophoresis was carried out at 300 mA and 20–25 V for 25 min. Slides were neutralized in a neutralization buffer (0.4 M Tris–HCl, pH 7.5) for 10 min before DNA precipitation in 95% ethanol for 10 min. DNA was stained with SYBR Gold (Invitrogen) and comets were imaged using a Nikon fluorescence microscope (Nikon, Melville, NY, USA). At least 50 cells were imaged and analyzed per slide using ImageJ (https://imajej.net) and OpenComet v1.3 plugin. Data are expressed as comparing the mean olive tail moment of cisplatin plus hydrogen peroxide with hydrogen peroxide-treated and untreated control samples, which represents the percentage of ICLs remaining on the fragmented DNA [15,16].

### 2.7. Slot Blot Assay for Intrastrand Adduct Repair

Following cisplatin treatment and time course for DNA repair, cells were collected and genomic DNA was isolated. DNA (2 μg) from corresponding cells was loaded into each well and vacuumed onto a nitrocellulose membrane. The membrane was incubated at 80 °C for 30 min, followed by blocking solution (5% milk in PBS-T) for 1 h and incubated overnight with α-Pt-GG antibody (1:1000, MABE416; clone ICR4; also referred to as CP9/19). The following day, the membrane was washed in PBS-T buffer four times for 15 min each before incubation with α-Rat secondary for 1 h. Membranes were washed for 5 min with 2 mL of combined ECL reagent before being imaged on ChemiDoc imaging system (Biorad, Hercules, CA, USA). Densitometry values of each blot were determined using ImageJ. Briefly, values for each band were measured and background-adjusted before standardizing to relative 0 time points. SYBR-Gold was utilized to stain DNA for normalization of DNA loading per well. The original slot blot images are represented in Supplemental Figure S5A,B.

### 2.8. In Vivo Study

A total of 36 female immunodeficient athymic NUDE mice (six mice per group) were purchased for this study from Envigo (Indianapolis, IN, USA). All animal procedures were conducted in accordance with protocols approved by the Wayne State University Institutional Animal Care and Use Committee. The mice were maintained under approved institutional animal care guidelines (protocol #: IACUC-19-06-1175; approved 9 September 2019). The mice were allowed to acclimate for 1 week before handling. Animals were housed under standard controlled environmental conditions with access to food and water ad libitum. Housing and husbandry were performed according to institutional animal care guidelines. Environmental enrichment was provided according to facility standard operating procedures. Mice were monitored regularly by research and/or veterinary staff. No previous procedures were performed before tumor implantation other than routine acclimatization and handling. The sample size of 6 mice per group was selected based on standard practice for preliminary in vivo xenograft efficacy studies and to provide sufficient power to detect biologically meaningful differences in tumor growth comparing a Polβ mutant to wildtype tumors while minimizing animal use. No formal a priori sample size calculation was performed. Tumor growth and treatment response was conducted using xenograft models established from MDA-MB-231 WT, E295K, and D256A cells. Tumors were generated by subcutaneous implantation of 5 × 10^6^ cells into the rear flank of each mouse. Once tumors reached an average volume of approximately 100 mm^3^, mice were randomized into treatment groups. Randomization was performed to distribute mice across groups and minimize imbalance in tumor volume at treatment initiation. To minimize potential confounding, treatment allocation was performed after tumor establishment, and tumor volumes were monitored using the same measurement method across all groups. Mouse body weight was measured regularly beginning on the day of tumor implantation and continued through the experimental endpoint. No additional strategies were used to control for cage location or order of treatment/measurement. Treatment groups were: WT + vehicle control, WT + cisplatin, E295K + vehicle control, E295K + cisplatin, D256A + vehicle control and D256A + cisplatin. Vehicle-treated mice received PBS, and cisplatin-treated mice received cisplatin at 3 mg/kg by intraperitoneal injection on days 12, 15, 19, and 25 after tumor implantation. Weight of the mice was measured regularly starting on the day of implantation and through the experimental endpoint. Once tumors became visible, volume was measured with calipers and defined as (width^2^ × length/2). Mice were monitored for tumor growth and general health throughout the study. The tumor volume was the primary outcome measure. Secondary outcome measures included mouse body weight and time to experimental endpoint. Mice were euthanized when tumor burden reached approximately 1000 mm^3^ or according to approved humane endpoint criteria. Animals were excluded from analysis only if tumors failed to establish, if tumor volume could not be reliably measured, or if there were technical complications unrelated to treatment, such as failed injection or other procedure-related issues. No animals, experimental units, or data points were excluded from the analysis. Mice were sacrificed once the burden of the tumor reached ~1000 mm^3^. Final tumor volumes were significantly lower in cisplatin-treated E295K and D256A groups compared with their corresponding vehicle controls, as determined by two-way ANOVA followed by post hoc multiple-comparison testing. Data are presented in the results as mean ± SD, with *n* = 6 mice per group. Statistical significance is indicated as * *p* < 0.05 and ** *p* < 0.01. For this hypothesis-testing study, tumor volume/tumor growth delay was the primary outcome measure. This study was not blinded, and thus, a limitation of the study is that there could be observer bias when measuring tumor volume. Group allocation was known to the personnel involved in treatment administration, animal monitoring, tumor measurement, and data analysis. Vehicle- and cisplatin-treated mice were handled and monitored using the same procedures, and tumor volumes were measured consistently by caliper throughout the study.

### 2.9. Vector Generation for CRISPR Knock-In of I260M

DNA oligonucleotides were purchased from IDT and used to clone the sgRNA into BbsI-digested pX330-U6-Chimeric_BB-CBh-hSpCas9 (a gift from Feng Zhang Addgene plasmid # 42230) [26]. The homology-directed repair template was cloned by Gibson Assembly into pFastBac Dual. Homology arms and a codon-optimized sequence containing the Polβ I260M mutation and the remaining C-terminal coding sequence was purchased from IDT as G blocks. These G blocks, a PCR-amplified puromycin resistance cassette, and a linearized pFastBac Dual backbone were cloned together by Gibson assembly. Gibson reaction product was then transformed into chemically competent bacteria and plasmid isolated using a Qiagen MiniPrep kit (Germantown, MD, USA). Correct assembly was validated by Sanger sequencing. A similar approach was utilized for Polβ E295K generation.

### 2.10. CRISPR-Cas9 Generation of Knock-In Clones

I260M knock-in cells were created via vectors generated in-house. Briefly, cells were transduced with pFastBac Dual vector containing the homology-directed repair sequence to knock-in the I260M mutation and a puromycin resistance cassette (downstream of the Polβ stop codon) as well as px330 containing Cas9 and the sgRNA. Three days post-transfection, cells were selected with puromycin before isolating clones. Once clones grew to adequate size, genomic DNA was harvested from cells and used alongside PCR primers flanking the genomic location of incorporation.

E295K knock-in cells were generated by the Sweasy lab similar to the approach used for I260M knock-in generation and DNA sequence verified to be homozygous knock-in in MDA-MB-231 cells. Briefly, cells were lysed in Lamelli buffer + β-mercaptoethanol, scraped and boiled for 15 min. at 90 °C. Protein fractions were analyzed by 10% SDS-PAGE and transferred to PDVF membrane. The membrane was then blocked with a 50 mL solution of 5% nonfat milk, 1× Tris-buffered saline (TBS; 50 mM Tris–HCl, pH 7.4, 150 mM NaCl), and 0.1% Tween 20 (TBS-T) for one hour at RT. After washing the blot twice with 1× TBS-T, primary antibody of Polβ (at a dilution of 1:1000; Abcam cat# ab26343) was added in a 5 mL solution of 5% nonfat milk and 1× TBS-T, and the blot was incubated overnight at 4 °C. Subsequent to being washed with 1× TBS-T solution three times, the membranes were washed twice with 1× TBS. The membranes were then incubated with anti-rabbit horseradish peroxidase-conjugated secondary antibody (at a dilution of 1:25,000, cat#NA9340V GE) for 1 h at RT. The membranes were then washed as described above. Proteins were visualized using an enhanced chemiluminescence kit used according to the manufacturer’s directions (ThermoFisher, Waltham, MA, USA). Actin-HRP antibody (Sigma-Aldritch, St. Louis, MO, USA, cat#A3854) at 1:10,000 in 5% BSA-TBS-t for 30 min, was used to normalize the protein loading. The levels of proteins were quantified by normalizing the levels to the housekeeping protein using Image Lab software (BioRad version 3.0.1). A limitation of the knock-in models could be the potential for off-target effects caused by CRISPR-Cas9 method albeit complementary approaches and additional mutant models show similar phenotypes and argue against this possibility.

### 2.11. Statistical Analysis

All experiments were performed with a minimum of three technical replicates accounting for a biological replicate and three separate biological replicates to generate data in each experiment unless noted otherwise. The analysis for IC50s for colony survival and MTS assays was similar to what was previously reported [15,16,17]. Data was summarized with means and standard deviations (mean ± SD) as indicated in the figure legends. Where appropriate, data was corrected for multiple testing, the distribution of data was checked for normality and a log transformation was applied where needed. Unpaired *t*-tests were used to distinguish differences between two groups and one-way ANOVA followed by post hoc analysis was used for groups of three or more. Tumor growth curves were analyzed by comparing tumor volumes among vehicle- and cisplatin-treated groups over time. For longitudinal tumor growth data, mixed-effects analysis was used, followed by appropriate multiple-comparison testing when applicable. Tumor volume comparisons at endpoints were performed using unpaired *t*-tests or one-way ANOVA with multiple-comparison correction, as appropriate. To further assess the robustness of the observed treatment effects, additional statistical analyses were performed. Since the tests for equal variance were significant, variance-robust analyses were used, and both Brown–Forsythe and Welch’s ANOVA confirmed significant differences among treatment groups. Pairwise Welch-corrected analyses further showed that cisplatin significantly reduced tumor burden in both mutant models compared with their matched vehicle controls, whereas WT cisplatin was not significantly different from WT vehicle. Together, these results show that the overall group differences were statistically significant even after accounting for unequal variances among treatment groups. Body weight was analyzed descriptively and/or statistically to assess treatment tolerability. Data are presented as mean ± SD. Statistical significance was defined as *p* < 0.05. Assumptions of the statistical tests, including approximate normality and variance distribution, were assessed where applicable. If data did not meet assumptions for parametric testing, appropriate non-parametric tests were used. All data analyses were carried out using the EZR package (R commander Version 2.8-0, R version 4.2.2, RStudio Inc., Boston, MA, USA) and SigmaPlot version 10.0.

## 3. Results

### 3.1. Sensitivity of Polβ Catalytic Mutants to Cis/Carboplatin

**Figure 1:**
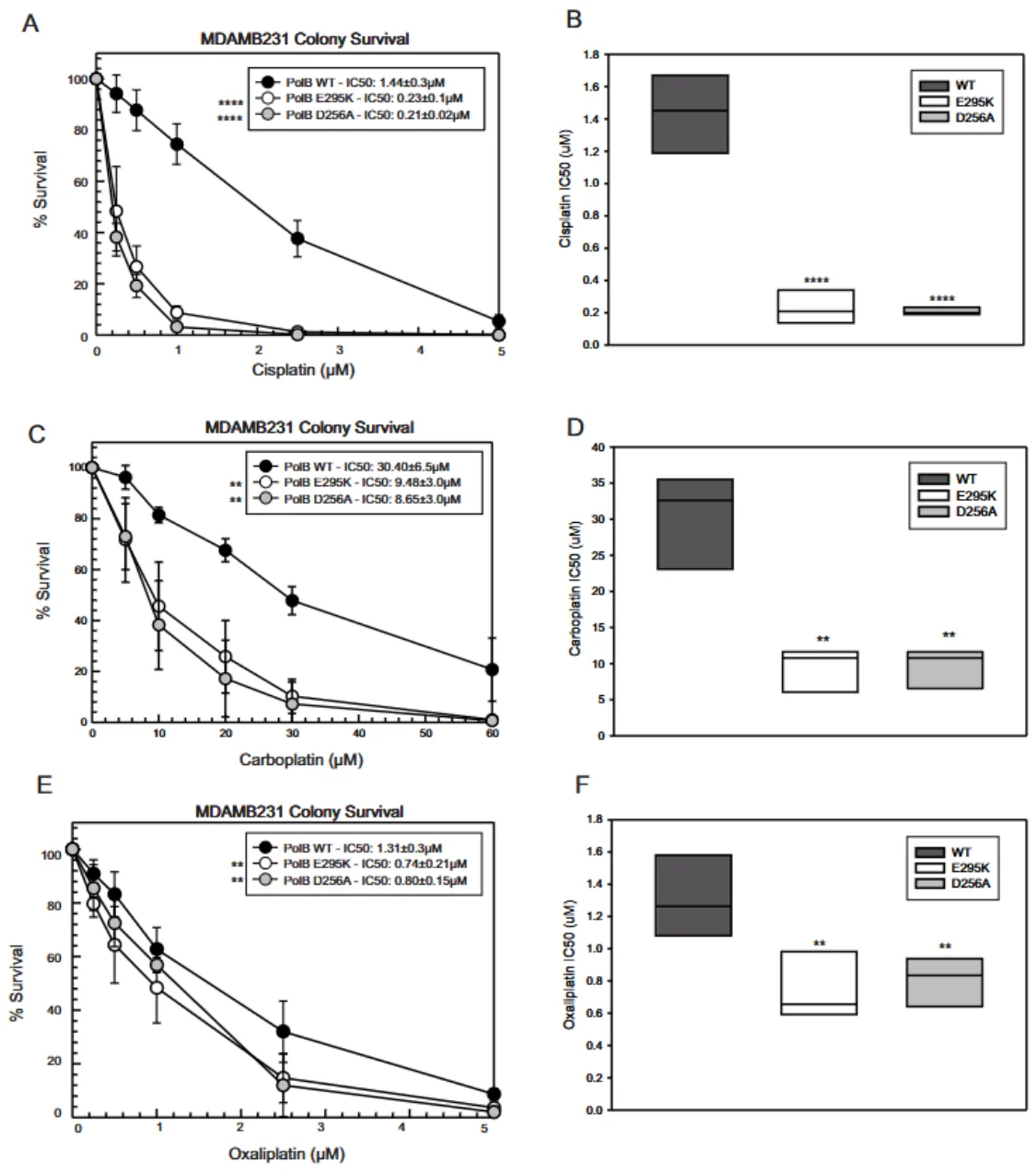
Catalytic-deficient polymerase beta induces selective platinum sensitivity. Colony survival assay of MDA-MB-231 cells treated for 2 h with (**A**) cisplatin, (**C**) carboplatin and (**E**) oxaliplatin. IC50 values plotted for cisplatin (**B**), carboplatin (**D**) and oxaliplatin (**F**). Data plotted as average ± SD of three independent replicates. IC50 values were calculated with CompuSyn software using a best fit of effect values at each dose and is presented as mean ± SD. IC50 values were compared to WT cells via a two-tailed *t*-test (** *p* < 0.01, **** *p* < 0.0001).

Our previous studies have demonstrated that knockdown of Polβ and other BER/MMR members led to a platinum-resistant phenotype while the knockdown of endogenous Polβ and re-expression of a catalytic dead Polβ mutation (D256A) showed a cisplatin-sensitive phenotype [15]. We sought to assess and compare another catalytic dead Polβ mutation, E295K, that was previously shown to have catalytic deficiency in a triple-negative breast cancer model [22]. A CRISPR-Cas9 knock-in approach was taken to homozygously express this mutation in MDA-MB-231 TNBC cells. These cells were confirmed for deficiency in BER activity by treating with alkylating agent Methyl Methanesulfonate (MMS) (Supplemental Figure S1A). To determine if this cisplatin-sensitive phenotype was a result of catalytic mutant Polβ, we first compared sensitivity to cisplatin (Figure 1A,B) and found similar sensitivity in both the D256A and E295K Polβ mutations. A limitation of the over-expressing D256A model is the levels of Polβ relative to endogenously expressed Polβ and the low potential that wildtype Polβ could be re-expressed. The normalized transcript expression of wildtype (WT), E295K homozygous clone, E295K heterozygous clone and the D256A over-expressing model are shown in Supplemental Figure S1B. The heterozygous E295K clone had an IC50 in the ~0.8 μM range compared with the ~0.22 μM for the E295K and D256A models. Additionally, we measured cell viability for these cells in an MTS assay treated with cisplatin and found a similar trend (Supplemental Figure S1C). There are differences observed in the IC50 values when comparing colony survival assays which monitor the ability of cells to replicate and grow into colonies of cells > 50 versus MTS assays which monitor metabolic readout for cell death. In accordance with prior research, we expected similar sensitivity to carboplatin due to the adducts formed being functionally identical to those induced by cisplatin. In these Polβ mutant models, we observe similar degrees of carboplatin sensitivity as compared with cisplatin (Figure 1C,D). In contrast, the ICLs induced by oxaliplatin do not form extrahelical cytosines to the same extent as cis/carboplatin, and thus, would not be expected to be recognized by APOBEC3 members, which induce cytosine deamination to uracil that are subsequently processed by the downstream BER pathway [15]. Thus, we did not initially expect to observe oxaliplatin sensitivity in these Polβ mutant models assuming drug sensitivity is driven solely by the non-productive processing of ICLs. Interestingly, we observed a modest enhanced oxaliplatin sensitivity in both the D256A and E295K Polβ mutant models (Figure 1E,F). These results suggest there are additional effects/processes potentially including blocking intrastrand adduct repair that may be impacted by catalytically deficient Polβ mutations that drive the drug responses in these models.

### 3.2. Reduced Rate of Adduct Repair in Polβ Catalytic-Deficient Clones

**Figure 2:**
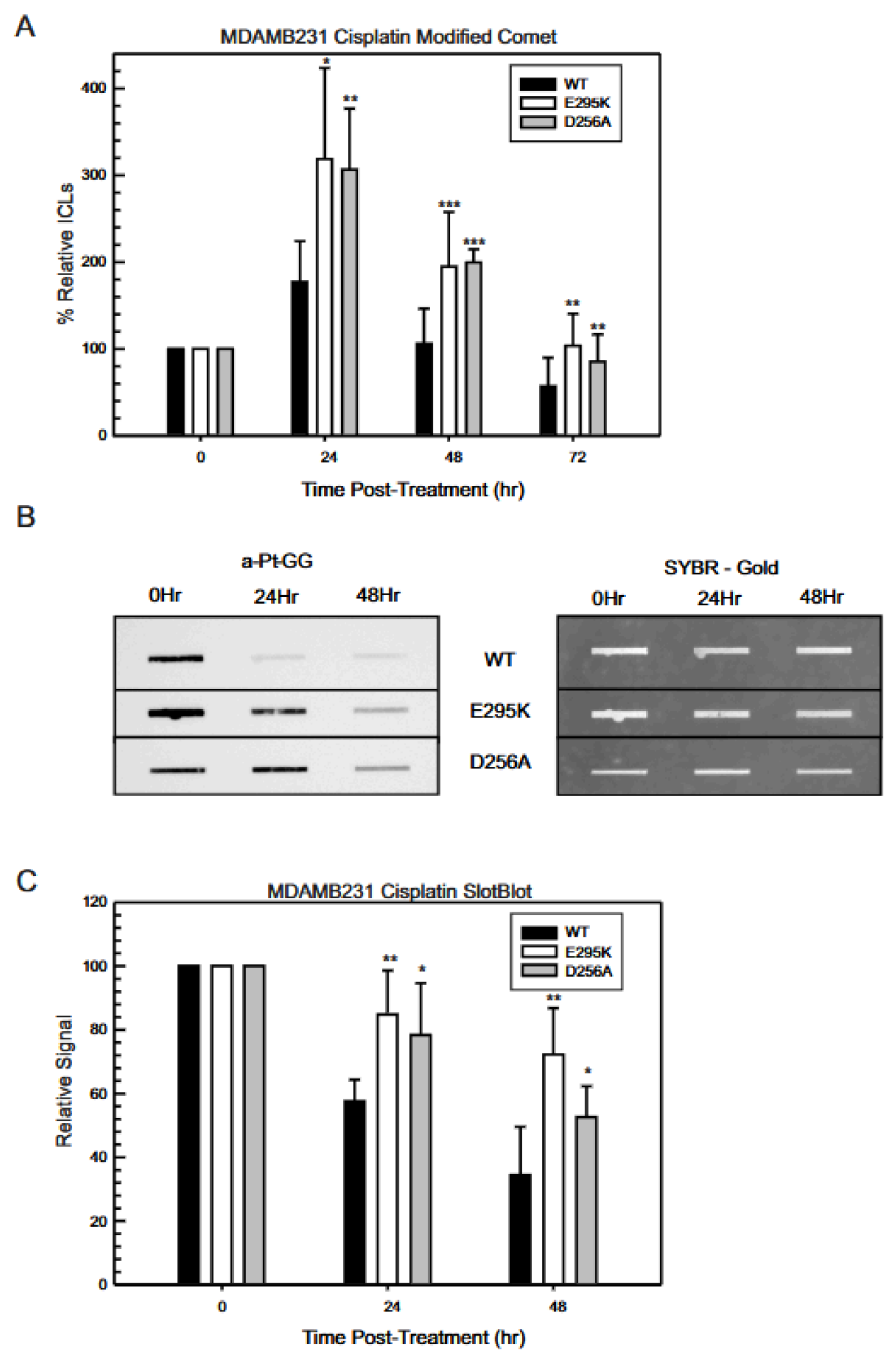
Both intrastrand adduct and ICL repair are inhibited in Polβ mutant-expressing cells. (**A**) Modified comet assay of cells treated with cisplatin (10 μM) for 2 h. Data is standardized to 0 h post-treatment time points and presented as mean ± SD of three replicates. (**B**) Slot blot assay against platinum GG intrastrand adducts of DNA collected from MDA-MB-231 cells treated with cisplatin (10 μM) for 2 h. (**C**) Band quantification of slot blot assay using ImageJ software. Data is standardized to 0 h post-treatment time point and is present as mean ± SD of three independent replicates. *p*-values were determined relative to WT sample using a two-tailed *t*-test (* *p* < 0.05, ** *p* < 0.01, *** *p* < 0.001). The uncropped blots are shown in Figure S5A.

In previous BER knockdown experiments, we found loss of this pathway to be associated with an increased rate of ICL DNA repair relative to wildtype (WT) expressing cells [15,16,17]. Therefore, we analyzed the rates of repair of the cisplatin-induced ICLs in our catalytic-deficient Polβ expressing cells via modified comet assays. We observed that the increased sensitivity is associated with a reduced rate of repair of the cis/carboplatin ICLs (Figure 2A). The accumulation and formation of ICLs peaks at ~18–20 h and thus, in all cases, there is an increase in ICLs from the time 0 to 24 h time points followed by repair of the ICLs from 24 to 72 h time points [15,16,17]. At 24 h, 48 h and 72 h post-treatment, there is a significant retention of ICLs in the Polβ E295K and D256A mutant cells compared with WT cells, demonstrating inefficient ICL DNA repair (Figure 2A). Previously published results demonstrated that the loss of BER pathway proteins had no significant impact on the repair of cisplatin-induced intrastrand adducts [16]. In these Polβ mutant models, however, utilizing an intrastrand adduct specific slot blot assay (antibody targets the primary GG intrastrand adduct), we demonstrate that the catalytic-deficient Polβ mutants also result in reduced rates of intrastrand adduct repair following cisplatin treatment (Figure 2B,C). These data suggest that catalytic-deficient Polβ drives cisplatin sensitivity through inhibition of both ICL and intrastrand adduct DNA repair.

### 3.3. Inhibition of Upstream BER Partially Restores Cis/Carboplatin Resistance

**Figure 3:**
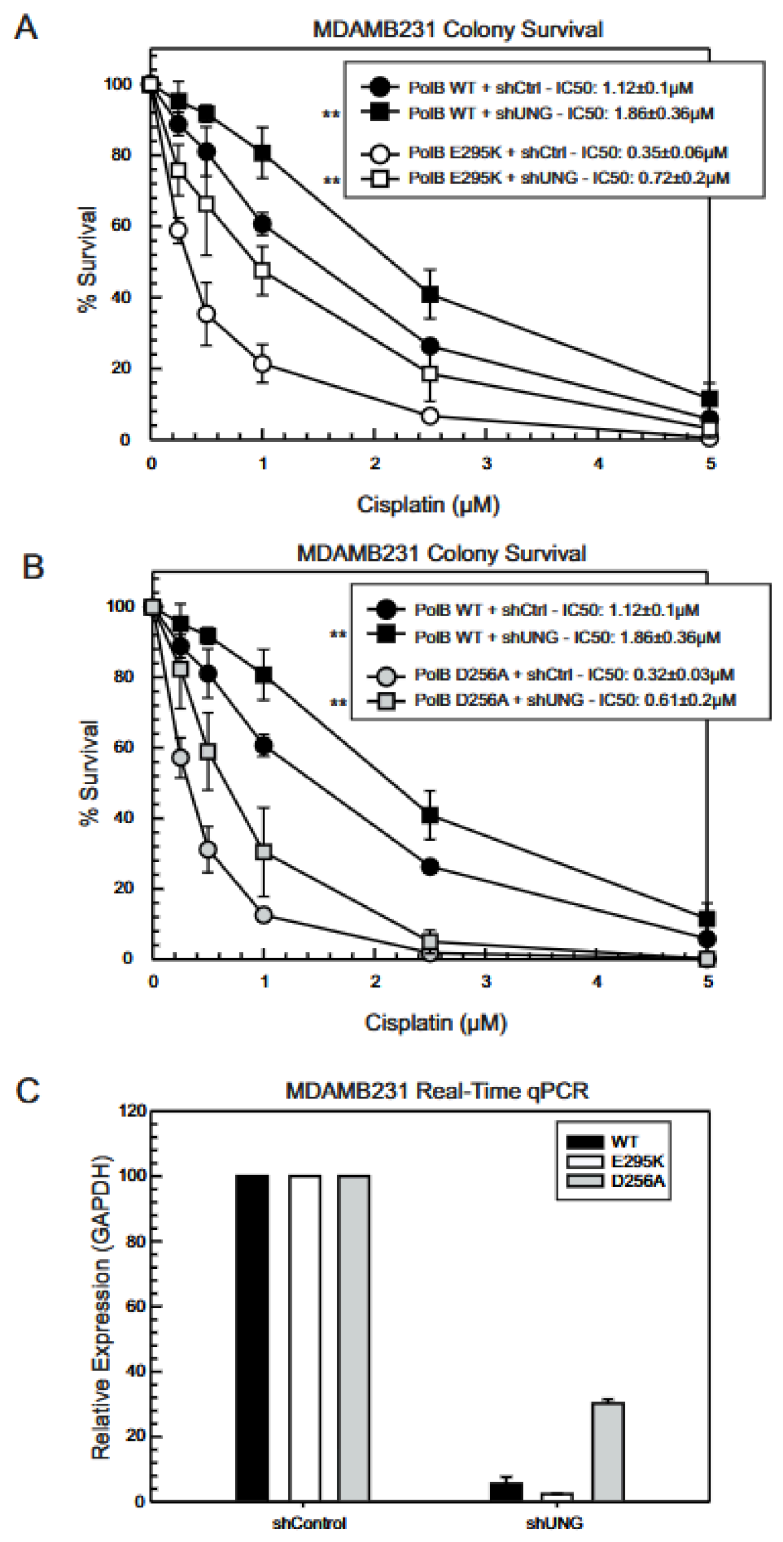
Inhibition of upstream UNG only partially induces resistance in catalytic-deficient Polβ cells. Colony survival assay of cells expressing either shCtrl or shRNA targeting upstream UNG were introduced in wildtype or (**A**) E295K and (**B**) D256A expressing cells and treated for 2 h with cisplatin in serum-free media. (**C**) Real-time-qPCR of UNG in shControl and shUNG expressing cells. Relative expression was acquired with GAPDH relative control, and expression was standardized to shControl values. Data plotted as average ± SD of three independent replicates. IC50 values were calculated with CompuSyn software using a best fit of effect values at each dose and are presented as mean ± SD. IC50 values and relative expression of shUNG were compared to WT via a two-tailed *t*-test (** *p* < 0.01).

**Figure 4:**
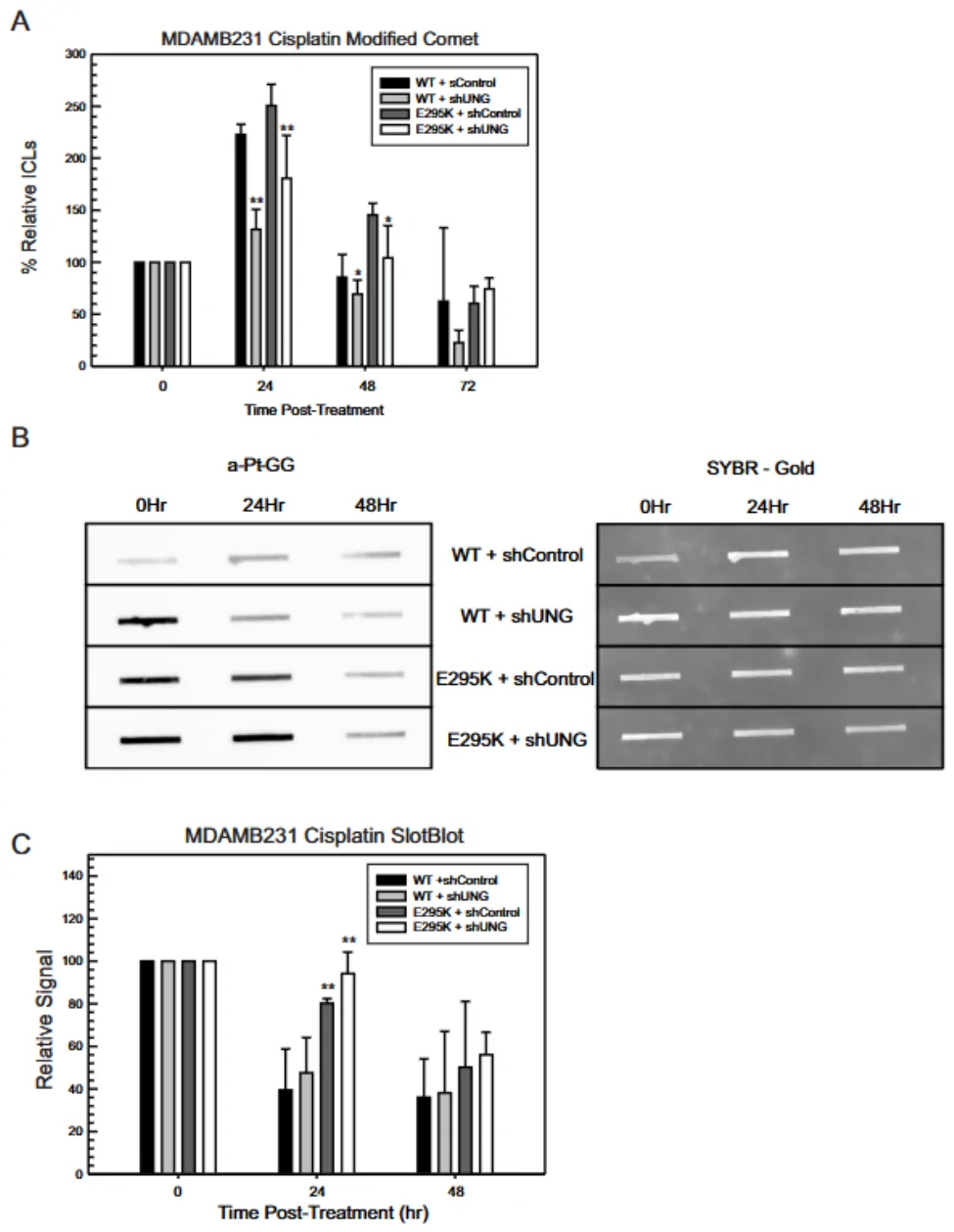
Inhibition of UNG results in increased ICL repair by not intrastrand adduct repair. (**A**) Modified comet assay of WT and E295K cells expressing either shControl or shUNG were treated with cisplatin (10 μM) for two h and assessed at 0, 24, 48, and 72 h post-treatment. Data was standardized to 0 h time point and plotted as average ± SD of three independent replicates. (**B**) Slot blot assay of WT and E295K expressing cells with shControl and shUNG were treated with cisplatin (10 μM) for 2 h and assessed for Pt-GG intrastrand adducts at 0, 25, and 48 h post-treatment. SYBR gold was used as a loading control. (**C**) Band quantification of slot blot assay using ImageJ software. Data plotted as average ± SD of three independent replicates. Comparisons at each time point were compared via a two-tailed *t*-test (* *p* < 0.05, ** *p* < 0.01). The uncropped blots are shown in Figure S5B.

We showed previously that loss of BER resulted in a cisplatin-resistant phenotype that was correlated with an increased rate of ICL DNA repair [15,16,17]. Here, to further define the mechanism(s) driving therapeutic response, we targeted multiple members of the BER pathway by shRNA knockdown and chemical inhibition. The results show resistance to cisplatin in WT MDA-MB-231 triple-negative breast cancer cells similar to what we previously observed (Figure 3A, WT vs. shUNG) [16,17]. If the drug sensitivity observed in the catalytic Polβ mutant models is solely attributed to the non-productive processing of ICLs, then knockdown of UNG would result in a complete reversal of the sensitive phenotype. The UNG knockdown experiments result in only partial reversal of the sensitivity of the Polβ catalytic E295K (Figure 3A, Ctrl vs. KD) expressing cells as well as those with D256A mutation (Figure 3B, Ctrl vs. KD) to cis/carboplatin. To ensure that incomplete reversal of cisplatin sensitivity was not due to insufficient UNG knockdown, we assessed transcript levels of UNG via RT-qPCR and found >90% knockdown in both WT and E295K expressing cells and >65% knockdown in D256A expressing cells (Figure 3C). This lack of complete resistance rescue was substantiated further with inhibition of APE1 incision by methoxyamine (MX) (Supplemental Figure S2A) as well as utilizing knockdown of APOBEC3 family members by shRNA (Supplemental Figure S2B,C) for both Polβ catalytic mutant models. To determine if there were effects on the repair of ICLs, we used the modified comet assay and found that knockdown of upstream UNG resulted in increased rates of ICL DNA repair in both WT Polβ expressing cells (Ctrl vs. UNG), as well as E295K expressing cells (Ctrl vs. UNG) (Figure 4A). UNG is upstream of Polβ recruitment and thus, if non-productive ICL DNA repair contributes to the sensitivity of these catalytic Polβ mutant models, then downregulation of UNG would increase ICL DNA repair. These results are consistent with that concept and highlight that non-productive ICL processing is contributing to some of the enhanced sensitivity in these Polβ mutant models. As expected, downregulation of the BER factors had no impact on the repair of intrastrand adducts as determined via slot blot assay in both WT expressing cells (Ctrl vs. UNG) and E295K expressing cells (Ctrl vs. UNG) (Figure 4B,C). The uncropped slot blots for Figure 2B and Figure 4B are shown in Supplemental Figure S5. These data are consistent with previously reported experiments where loss of BER pathway members was observed with only an increased rate of ICL repair [15,16,17].

### 3.4. Reduced Fidelity Polβ Clones Also Display Platinum Sensitivity

**Figure 5:**
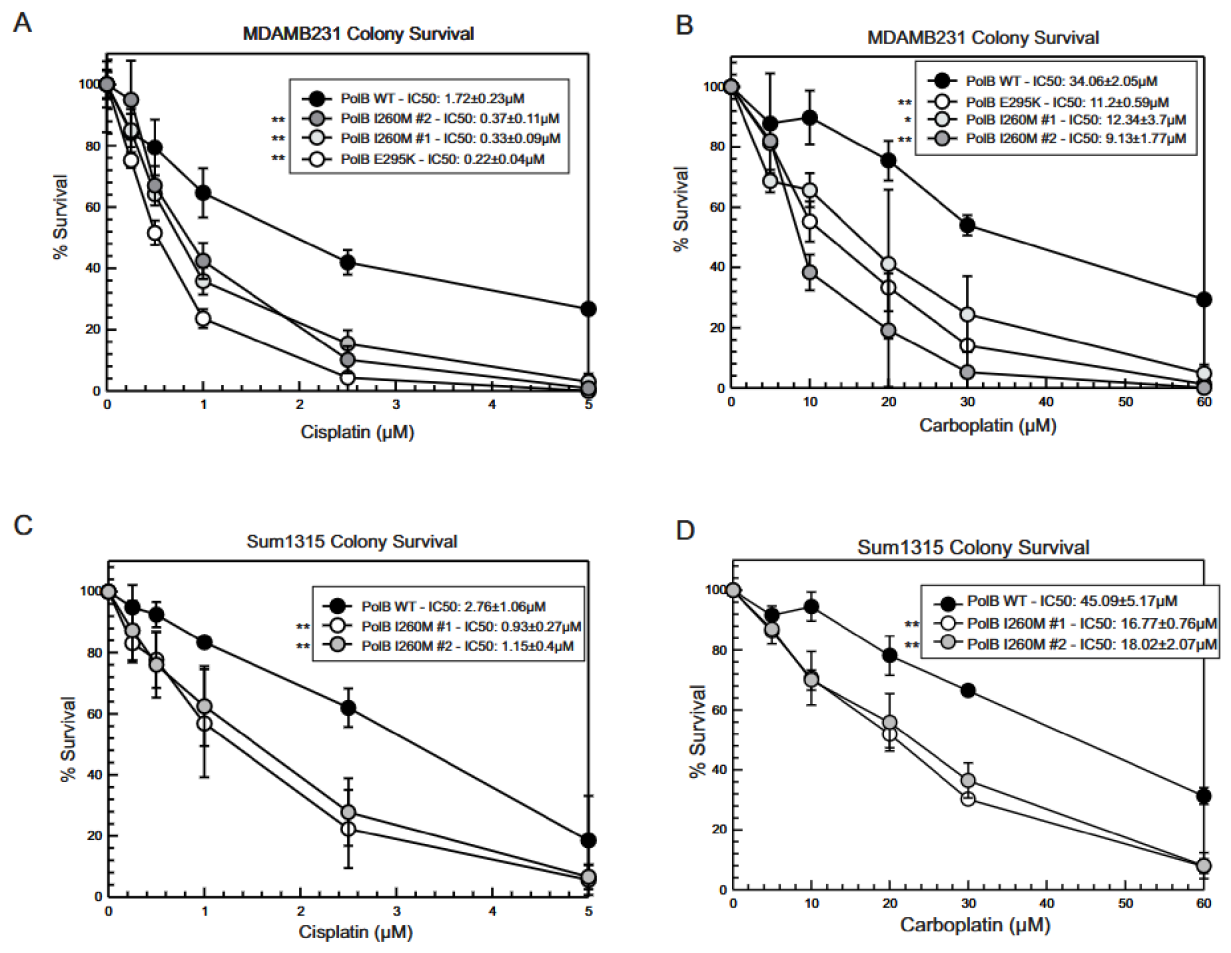
I260M induces cisplatin and carboplatin sensitivity in MDA-MB-231 and SUM1315 cells. Colony survival assay of MDA-MB-231 cells expressing either Polβ WT, E295K, or CRISPR knock-in clones expressing I260M variant were treated for 2 h with (**A**) cisplatin or (**B**) carboplatin. Colony survival assay of SUM1315 cells expressing either Polβ WT or CRISPR knock-in clones expressing I260M variant were treated for 2 h with (**C**) cisplatin or (**D**) carboplatin. IC50 values were calculated with CompuSyn software using a best fit of effect values at each dose and is presented as mean ± SD. IC50 values were compared to WT cells via a two-tailed *t*-test (* *p* < 0.05, ** *p* < 0.01).

To gain further clinical implications, we tested another Polβ mutant (I260M) in our cell line models. The I260M mutation is associated with some polymerase catalytic deficiency as well as a mutator phenotype based on its reduced fidelity (i.e., more likely to insert the incorrect nucleotide) [27]. To accomplish this, we first looked at the expression of Polβ in several breast cancer cell lines as seen in Supplemental Figure S3. To incorporate this mutation, we utilized a CRISPR-Cas9 knock-in methodology. The advantage of this was the ability to insert a puromycin selection cassette to enrich for edited cells. We isolated clones from puromycin-resistant cell lines, isolated genomic DNA, and screened for knock-in by PCR (Supplemental Figure S4). Of note, we were unable to identify clones with homozygous knock-in of the I260M mutation. There are several potential underlying reasons for this, one speculative possibility is that the expression of this mutant in a homozygous manner is too toxic for cells to tolerate. Further studies would need to be conducted to fully assess the reason for generation of only heterogeneous clones. Previous work has shown other mutations of Polβ to be present in a dominant negative fashion [22,27], highlighting the overall impact that a single allelic variant can have for this protein. Next, we tested several of these clones for cisplatin and carboplatin sensitivity and found similar levels of sensitivity as compared with the E295K and D256A expressing cells when expressed in the MDA-MB-231 triple-negative breast cancer cell line model (Figure 5A,B). We also managed to knock-in the I260M variant in another TNBC cell line model (SUM1315) and found that this mutation also sensitized these cells to cisplatin and carboplatin compared to WT cells (Figure 5C,D); however, the drug sensitivity was at a lower fold difference relative to MDA-MB-231 cells. One likely reason for this reduced fold difference in sensitivity is that SUM1315 express Polβ at lower levels relative to the MDA-MB-231, thus having a lower amount of Polβ to inhibit the repair of cis/carboplatin induced adducts (Supplemental Figure S3). These data support that both catalytic and fidelity Polβ mutations can inhibit the repair of cisplatin and carboplatin-specific adducts and drive sensitivity to these drugs. Future studies with the I260M mutant model will be needed to resolve whether the catalytic activity specifically or the fidelity deficiency of Polβ in this mutant drives the cis/carboplatin sensitivity. The results of different Polβ mutants on cis/carboplatin sensitivity could be impacted by gene dosage for a given mutation, as the I260M mutant model is only a heterozygote, versus E295K and D256A being homozygous. In the instance of gene dosing, the amount of mutant Polβ protein present could have a significant impact on cis/carboplatin response. Additionally, the mechanism(s) related to cis/carboplatin sensitivity could be rate limited by other upstream processing events such that the relative amount of mutant Polβ plays less of a role or possibly that specific Polβ mutations could be dominant negative even in heterozygous models. The I260M mutant models display IC50 values similar to those of E295K and D256A models. Further studies will be needed to fully understand whether gene dosing for a specific Polβ mutant can impact drug response or whether certain Polβ mutants are dominant negative in mediating cis/carboplatin response.

### 3.5. Polβ Catalytic-Deficient Clones Induce Tumor Growth Delay with Cisplatin Treatment

**Figure 6:**
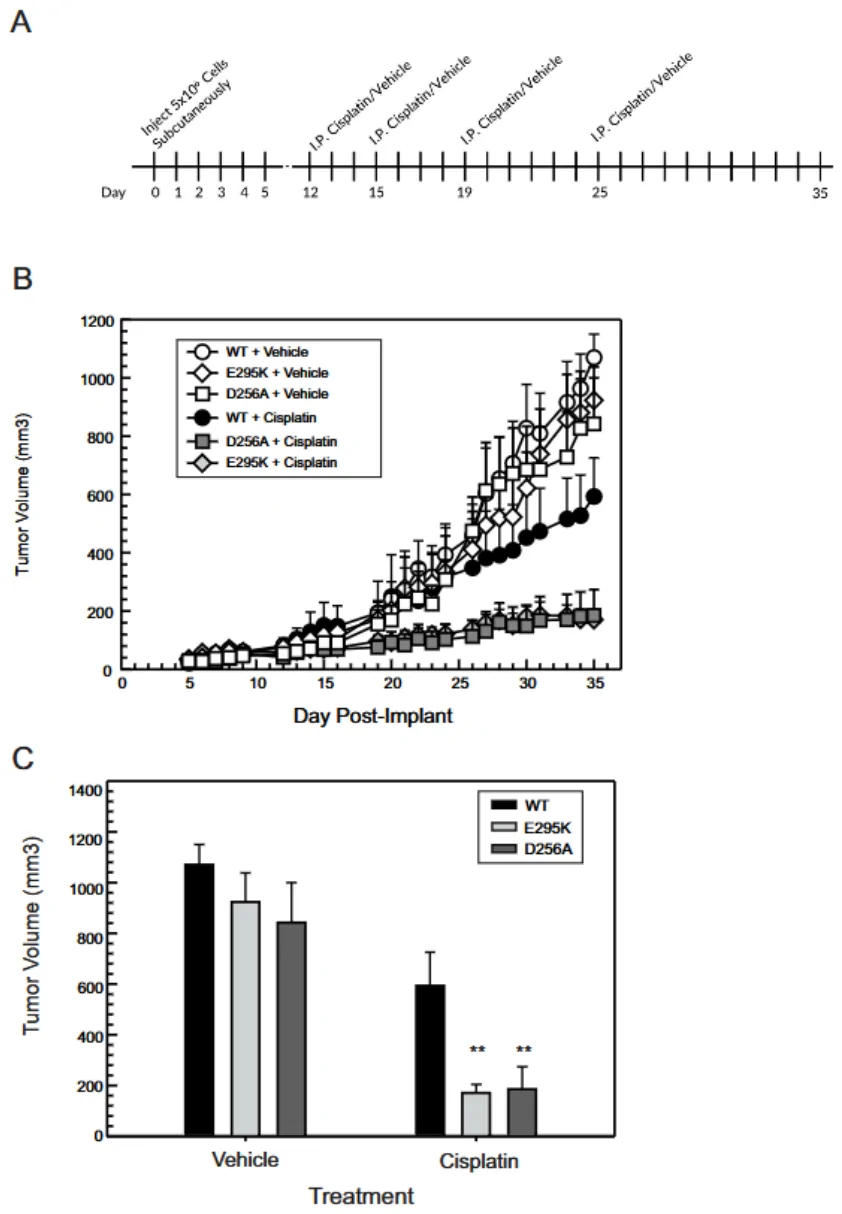
Catalytic-deficient Polβ drives a robust response to cisplatin in an in vivo mouse xenograft model. Mice were implanted with either WT, E295K, or D256A expressing cells and treated with vehicle or cisplatin. (**A**) Treatment scheme for the study. (**B**) Tumor volume was monitored and final tumor volumes were reported over time. Data were plotted as the average ± SD of each group (6 mice per group). (**C**), Final tumor volumes were compared using a two-way ANOVA and post hoc test (** *p* < 0.01).

Based on our previous in vitro data suggesting that catalytic deficiency of Polβ results in cisplatin sensitivity, we assessed whether these mutations would result in greater tumor growth inhibition in an in vivo mouse model. To accomplish this, we implanted MDA-MB-231 cells (WT, D256A, or E295K) subcutaneously into the flanks of nude mice to establish xenograft tumor models. Once tumors were established (~75 mm), we randomly assorted mice into either vehicle- or cisplatin-treated groups (controlling treatments and cell-type for average tumor size) and followed the treatment scheme outlined in Figure 6A. Briefly, mice were treated with either saline-solution or cisplatin solution (3 mg/kg) via intraperitoneal injection. Mice were monitored for body weight loss during the experiment and displayed no signs of toxicity. The mice harboring tumors from catalytic-deficient Polβ cells were found to have a significant tumor growth delay compared to those with WT expressing cells and harbored significantly smaller tumors at the termination of the study (Figure 6B,C, WT vs. E295K vs. D256A). We have utilized two separate Polβ catalytic-deficient models (E295K knock-in and re-expressed D256A) in MDA-MB-231 cells to assess response to cisplatin, but a limitation of the studies is that the genetic background in this model could impact therapeutic response. This highlights the need to further assess Polβ mutants in other models. This data, however, provides clear evidence that cisplatin-based therapies could be potentially beneficial to those patients harboring tumors with catalytic-deficient Polβ.

## 4. Discussion

**Figure 7:**
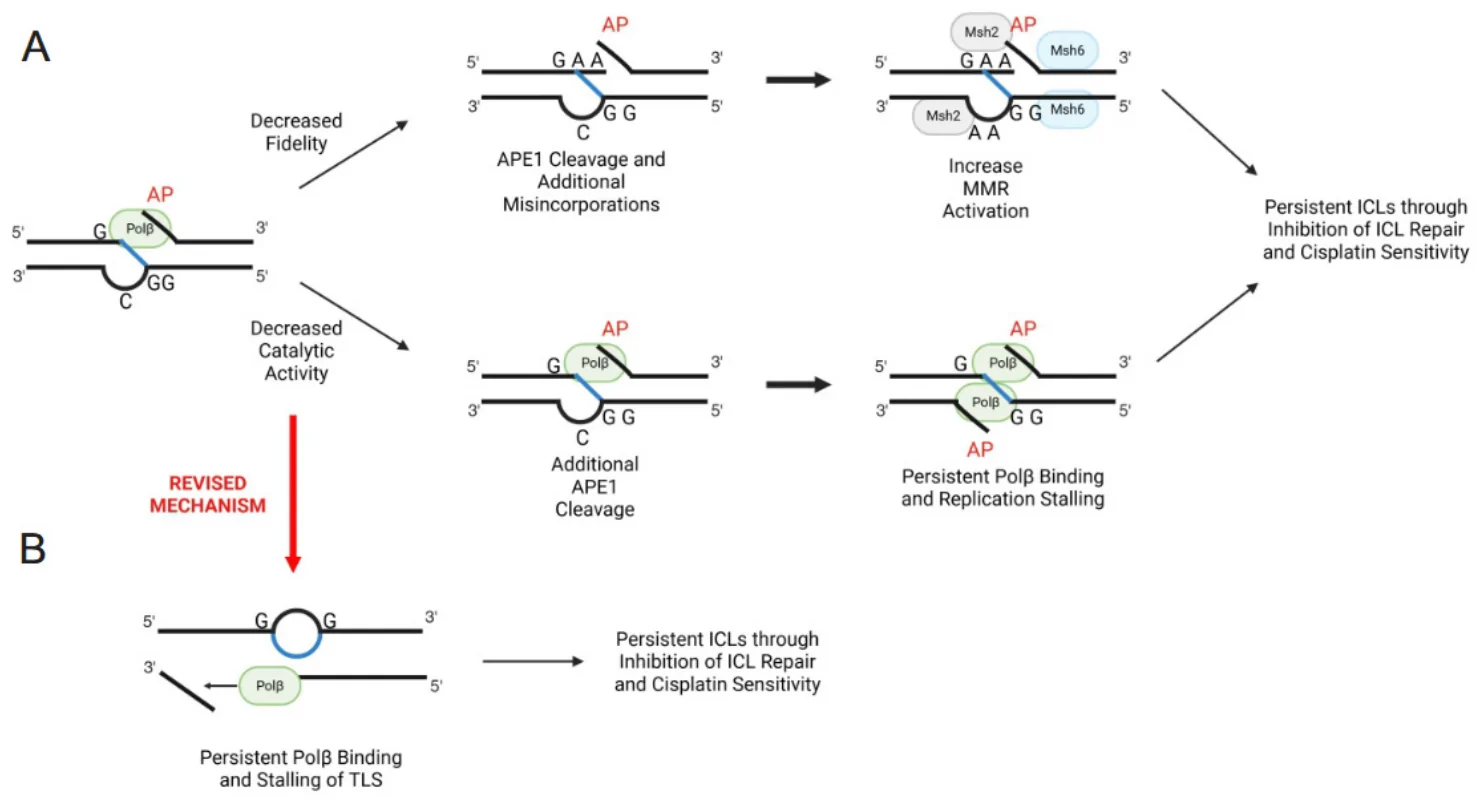
Proposed mechanistic model of mutant Polβ sensitivity to cis/carboplatin. The cells expressing mutant Polβ are shown to be selectively sensitive to cisplatin and carboplatin treatments. We show that this sensitivity is associated with a reduced rate of both intrastrand adduct and ICL repair induced by these drugs. (**A**) In regard to the ICL response, we hypothesize that Polβ is able to bind to these ICL sites as a consequence of APOBEC3 family member deamination of extrahelical cytosines [15]. The deamination of cytosine to yield uracil flanking the ICL is subsequently removed by UNG. APE1 is recruited and incises 3′ of the ICL to release the abasic site (AP) which enables Polβ recruitment. Catalytic-deficient Polβ mutations can stall the polymerase at the ICL and block productive repair, while Polβ fidelity mutants can result in introduction of the wrong nucleotides at the ICL site and drive more mismatch repair (MMR) protein, which, similar to catalytic mutants, can lead to persistent ICLs and cis/carboplatin sensitivity. In (**B**), both catalytic and fidelity Polβ mutations can also impact the translesion synthesis (TLS) past an intrastrand adduct (indicated by red arrow) and also block NER access and repair of these lesions to contribute to cis/carboplatin drug sensitivity. This is a speculative mechanistic model based on supporting data but needs more in-depth experiments to fully understand the mechanisms driving cis/carboplatin response.

In this study, we show a cis/carboplatin-specific sensitivity induced by several mutations of Polβ. These mutations affecting polymerase catalytic activity and fidelity led to a reduction in the repair of both intrastrand adducts as well as ICLs (Figure 7; proposed mechanistic models). We show that this increased sensitivity is partially impacted by downregulation or inhibition of upstream BER factors that would normally induce cis/carboplatin resistance via non-productive ICL processing. We specifically show that these Polβ mutations inhibit the repair and removal of both intrastrand adducts and ICLs. Additional work has shown that an increased catalytic phenotype mutant D160G also results in an increased cisplatin sensitivity [28]. This increased sensitivity was also associated with a reduced rate of repair in intrastrand adducts, in part through inhibition of XPA binding. Interestingly, the P242R Polβ mutation, which is associated with partial loss of catalytic activity, results in cisplatin resistance [20]. The authors show that the P242R Polβ mutation results in an XPA interaction which likely stimulates NER to repair the intrastrand DNA adducts [20].

These results highlight the importance of investigating the various clinically relevant Polβ mutations to fully understand which can mediate the response to platinum-based chemotherapy. To fully appreciate the potential clinical usage of Polβ mutations in being considered in genomic screening assays for predicting platinum-based chemotherapy response, more studies need to be conducted. There is some additional work reviewing the possibility of machine learning in helping to determine which mutations would be useful in predicting treatment courses [29]. The variety of mutations and ultimate loss or gain of phenotype in the different domains of Polβ drive the need for continued research into the mechanism of how this protein works in multiple settings. The variety in phenotypes also suggests that crystallography of Polβ-DNA interactions with the addition of platinum adducts could be useful in determining residues critical at these sites. Overall, our data suggests that Polβ interacts non-productively at both cisplatin intrastrand and ICL DNA adducts, helping to drive a sensitive phenotype.

The xenograft in vivo data highlight the potential tumor response to cisplatin in these Polβ mutant models. The significant tumor growth delay in these Polβ mutant models further highlights the potential use of these mutations in the clinical setting to help predict and drive better patient responses to specific types of therapy, including cis/carboplatin. The potential use of Polβ mutants as biomarkers would need more comprehensive assessment before being put into practical use. Treatment for patients consists of several weeks and is primarily used in a neoadjuvant setting in stage 4 TNBC [30]. Loss of Polβ has been shown to be a driver in mutagenesis and tumorigenesis [22,31]. Thus, targeting patients whose tumors harbor Polβ catalytic or fidelity mutations with multiple rounds of platinum therapy should lead to more robust and durable tumor responses and potentially these tumor-specific mutations could lead to better overall patient survival.

## 5. Conclusions

This study revealed that Polβ E295K, D256A and I260M mutations in the MDA-MB-231 TNBC cell line model can drive a therapeutic sensitivity to cis/carboplatin. The sensitivity of Polβ E295K and D256A mutant models to cis/carboplatin treatment is mediated through reduced repair of both intrastrand adducts as well as ICLs. Targeting upstream protein factors in the BER pathway, which plays a role in non-productive ICL repair, did not fully rescue a cis/carboplatin-resistant phenotype as is observed with Polβ knockdown. These results further support a contribution of both intrastrand adducts and ICLs in the sensitive phenotype of Polβ catalytic mutant models. This is an important finding for the field in understanding which lesions are responsible for mediating the sensitive phenotype in the Polβ mutant models. Potential mechanisms driving the sensitivity are proposed and discussed in Figure 7. This proposed mechanistic model is supported by the data in this report, but more in-depth experiments would be needed to fully decipher the mechanisms driving cis/carboplatin response in Polβ mutant models. Specifically, the potential impact of gene dosing and the possibility that different Polβ mutants could have dominant negative activity to drive cis/carboplatin efficacy need to be more thoroughly resolved. Additionally, tumor growth delay was observed in both E295K and D256A Polβ mutant xenograft models compared with wildtype control cells. These studies highlight the potential to utilize cancer-specific Polβ mutations in cancer patients to stratify prior to treatment decisions; however, more comprehensive analysis would be needed to fully validate the Polβ mutants for use in the clinical setting, including assessment of patient samples and drug response outcomes.

## Figures and Tables

**Table 1:** RT-PCR primers.

Target	Forward Primer	Reverse Primer
GAPDH	CGCTCTCTGCTCCTCCTGTT	CCATGGTGTCTGAGCGATGT
UNG	AGAAGGGCAGTGCCATTGATAGG	TCAATGGGCTTCTTGCCAGACTTCT

## Data Availability

Data generated from this study can be shared with appropriate investigators with request to the corresponding author.

## Supplementary Materials

The following supporting information can be downloaded at: https://www.mdpi.com/article/10.3390/cancers18162531/s1, Figure S1: Polβ catalytic-deficient mutants results in BER Deficiency and Display Sensitivity to Cisplatin; Figure S2: Inhibition of upstream APE1 and APOBEC3 only partially induces resistance to cisplatin; Figure S3: Protein Expression of Polβ in breast cancer cell lines; Figure S4: PCR Demonstrates Incorporation of I260M Allele via Homology Template. Figure S5: Original slot blots from main figures. (A), original slot blot Figure 2B; (B), original slot blot Figure 4B.
