# Supplementary material for: DNA Polymerase Beta Catalytic and Fidelity Mutations Drive Platinum-Specific Drug Sensitivity
Source: Cancers (Basel). 2026 Aug 7;18(16):2531. doi: 10.3390/cancers18162531 (PMC13511097; doi:10.3390/cancers18162531)

Supplemental Data

Supplemental Figure S1: Polβ catalytic deficient mutants results in BER Deficiency and Display Sensitivity to Cisplatin. (A) Colony survival assay of MDA-MB-231 cells expressing either WT Polβ or E295K variant were treated for 4 hours with alkylating agent MMS in serum free media. (B) Normalized qPCR assessment of WT, E295K homozygous, E295K heterozygous and D256A mutant models (C) MTS viability of MDA-MB-231 cells treated with cisplatin for 2hrs and assessed 72hours later. Data plotted as average ± SD of three independent replicates. IC50 values were compared to WT via a two-tailed t-test (* $p<0.05$, **$p<0.01$, ***$p<0.001$, ****$p<0.0001$).

Supplemental Figure S2: Inhibition of upstream APE1 and APOBEC3 only partially induces resistance to cisplatin. (A) Colony survival assay of MDA-MB-231 cells expressing either WT Polβ or E295K variant were pre-treated with 20μM of APE1 inhibitor methoxyamine for 4 hours before treatment with cisplatin for 2hours in serum-free media. (B and C) Colony survival assay of MDA-MB-231 cells expressing either shCtrl or shRNA targeting Apobec3 members were introduced in Polβ Wild-Type and B) E295K and C) D256A expressing cells. Data plotted as average ± SD of three independent replicates. IC50 values of shUNG were compared to shControl via a two-tailed t-test (* $p<0.05$, **$p<0.01$, ***$p<0.001$, ****$p<0.0001$).

Supplemental Figure S3: Protein Expression of Polβ in breast cancer cell lines. Protein was collected from breast cancer cell lines and western blot conducted using an anti-Polβ antibody and β-Actin loading control.

Supplemental Figure S4: PCR Demonstrates Incorporation of I260M Allele via Homology Template. Genomic DNA from individual cell colonies grown from puromycin selected CRISPR knock-in pools were assessed for incorporation of puromycin resistance vector via PCR amplification using primers flanking incorporation site.

Supplemental Figure S5: Original slot blots from main figures. (A), original slot blot Figure 2B; (B), original slot blot Figure 4B.

Supplemental Figure S1

A

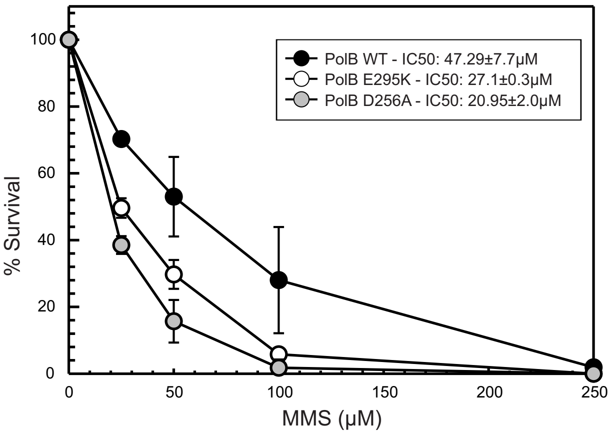

B

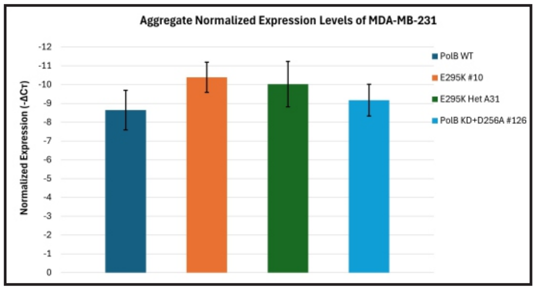

C

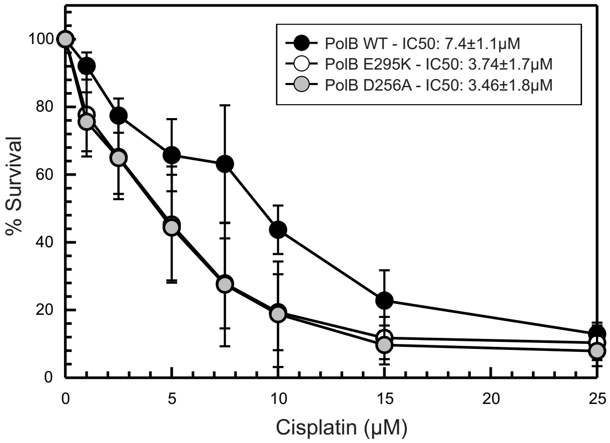

Supplemental Figure S2.

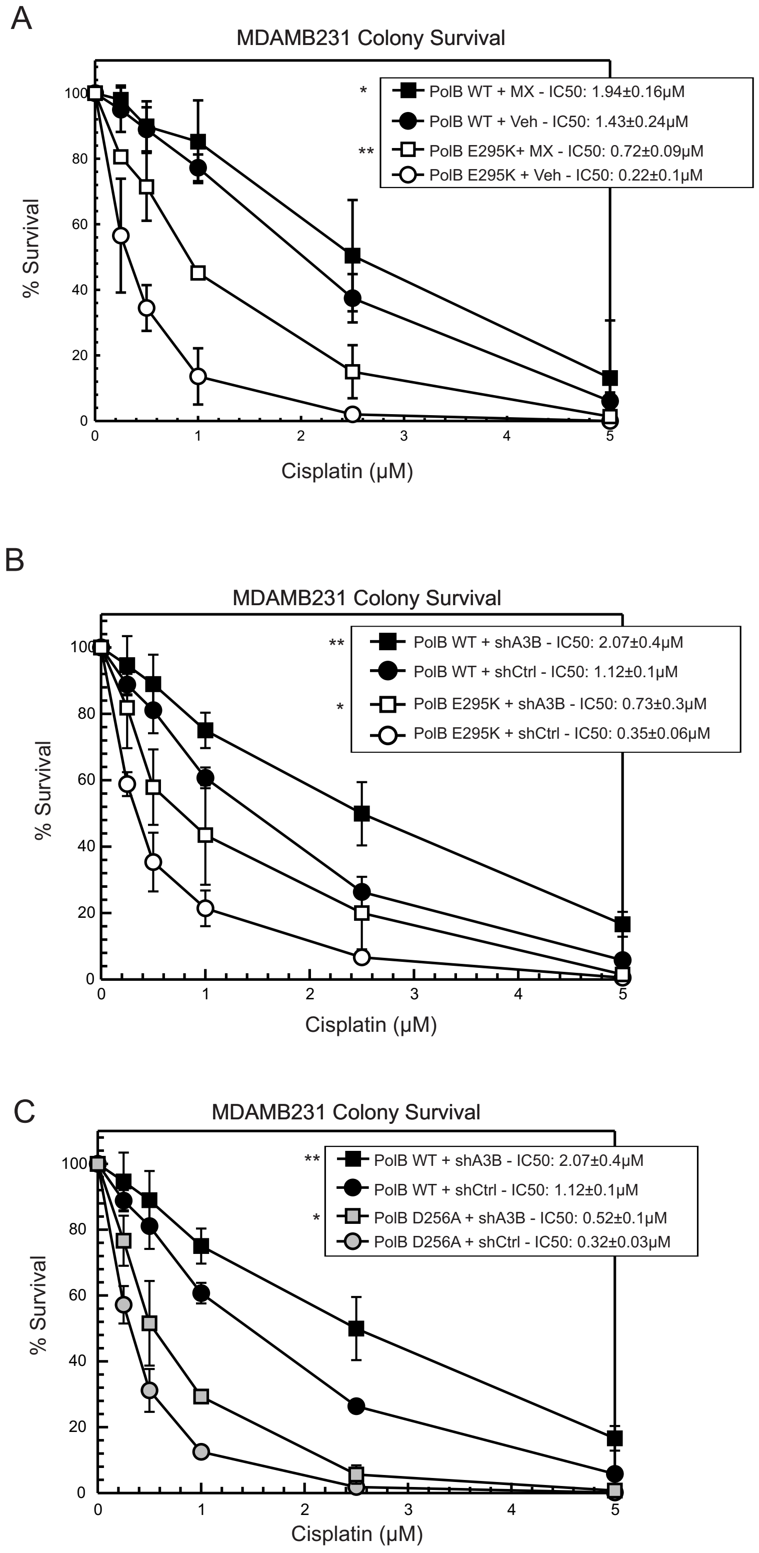

Supplemental Figure S3

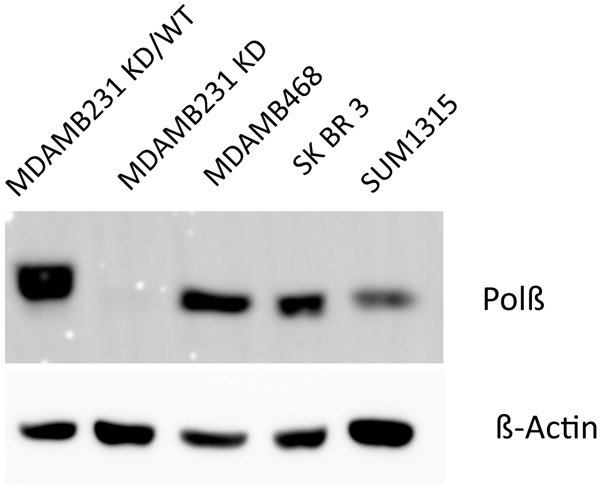

Supplemental Figure S4

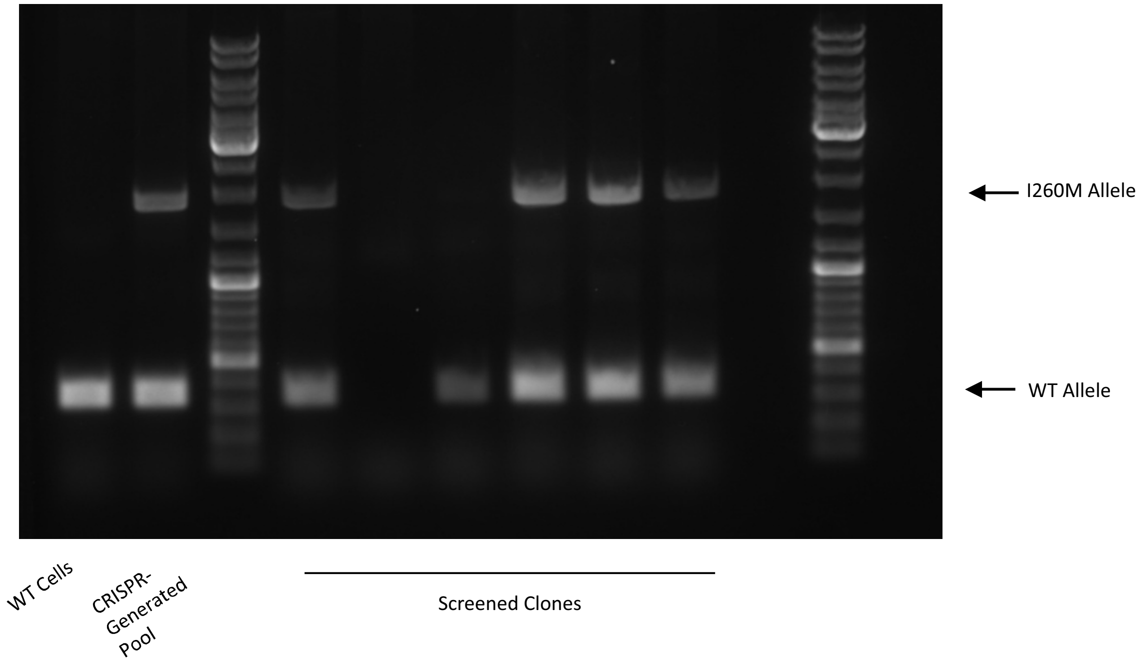

Supplemental Figure S5

A

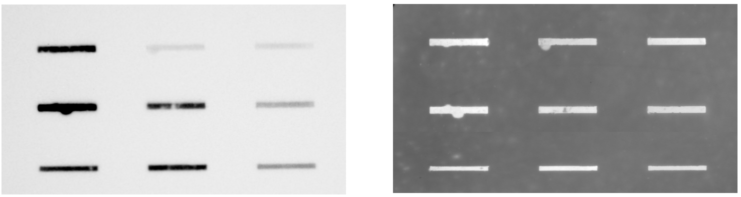

B

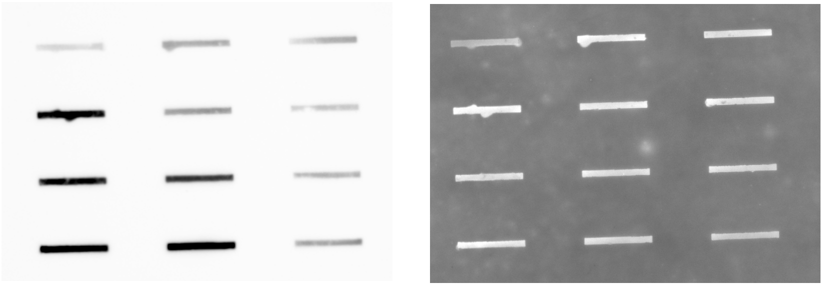

Supplement: Supplementary file 1 [file cancers-18-02531-s001.zip › cancers-4409939-supplementary.pdf]
